# Parity and mode of birth and their relationships with quality of life: A longitudinal study

**DOI:** 10.1371/journal.pone.0273366

**Published:** 2022-09-09

**Authors:** Deborah L. Davis, Chunsen Wu, Wendy J. Brown, Ellen A. Nohr

**Affiliations:** 1 Faculty of Health, University of Canberra, Canberra, ACT, Australia; 2 ACT Government Health Directorate, Canberra, ACT, Australia; 3 Department of Clinical Research, Research Unit for Obstetrics and Gynaecology, University of Southern Denmark, Odense, Denmark; 4 School of Human Movement and Nutrition Sciences, University of Queensland, St Lucia, QLD, Australia; 5 Faculty of Health Sciences and Medicine, Bond University, Gold Coast, Australia; Flinders University, AUSTRALIA

## Abstract

**Objective:**

To examine how (a) parity and (b) mode of birth were associated with later Quality of Life (QOL) in young adult women, with a mean follow-up of 11.0 years.

**Design:**

Prospective cohort study

**Setting:**

Australia

**r sample:**

A total of 7770 women participating in the 1973–1978 cohort of the Longitudinal Study of Women’s Health.

**Methods:**

Linear regression models were used to estimate (1) prospective associations between parity and mode of birth with eight subscale and two summary scores of the SF36, assessed after a mean follow-up of 11 years., and (2) differences between SF36 scores at follow up for women in different parity and mode of birth categories.

**Main outcome measure:**

Quality of Life as measured by the SF36

**Results:**

Women experiencing no births (parity 0) and one birth (parity 1) had lower scores on all the physical health measures, and on some mental health measures, than women who had 2 births (parity 2) (all p<0.05).

**Conclusions:**

Parity and mode of birth may have long-term implications for women’s physical and mental health. Both childless and women with only one child had poorer physical and mental health than their peers with two children. Women with only caesarean section(s) also had poorer health than women who had vaginal birth/s.

## Introduction

Childbearing is a profound event, with potential to impact not only physical, but emotional, psychological, and social aspects of life. The effects of having a baby on quality of life (QOL) in the years following a birth have not however been widely studied. Although childlessness, which can be voluntary, involuntary and/or circumstantial [1], has physical and mental health consequences that may resonate in the personal and social spheres [2], few studies have examined the effects of number of children on later QOL. Research has shown that childless women have poorer physical and mental health than women with children during the childbearing years [2], and experience social exclusion in a national context that is described as pronatalist [3].

For women who do have children, mode of birth may also have lasting physical and mental health effects. However, most studies have examined only clinical or physical health outcomes [4], and few have used patient based measures of health which consider a broader range of outcomes, including quality of life [5]. Very few studies in this field have had a follow-up of more than 12 months postpartum with most 6-months or less [6–11] and occasionally up to 12 months [12, 13].

Most studies of mode of birth have focussed on caesarean section, which almost doubled in prevalence between 2000 and 2015 [14] and now accounts for 27% of births in high income countries globally and 33.5% in Oceania [15]. Indeed, according to the World Health Organization, it is the most common major surgery performed around the world [16]. Caesarean sections [13] have been implicated in poorer short term QoL [13, 17] but few studies have measured QoL after more than one year. One challenge in this field is that these associations may vary according to whether the caesarean section is conducted for emergency or medical indications, rather than on request [18].

Very few studies have compared the longer term effects on QoL in women who have vaginal births with those who experience instrumental birth or caesarean section, and none has considered the effects of childlessness and parity on QoL in the longer term. The aim of this study was therefore to examine associations between (a) parity and (b) mode of birth, with QoL, drawing on data collected over 15 years by the Australian Longitudinal Study of Women’s Health.

## Methods

### Data sources and participants

Data for this study were from the 1973–78 cohort of the Australian Longitudinal Study on Women’s Health. The women were randomly selected from a national database of all Australian permanent residents born between 1973 and 1978. Respondents completed the first survey (S1) in 1996 (*n* = 14,247) when they were 18–23 years, with follow-ups in 2000 (S2, 22–27 years), 2003 (S3, 25–30 years), 2006 (S4, 28–33 years), 2009 (S5, 31–36 years), 2012 (S6, 34–39 years), and 2015 (S7, 37–42 years). Recruitment details, baseline characteristics, response and attrition rates have been reported elsewhere [19].

At the time of recruitment, the women were largely representative of 18–23-year-old women in Australia, but over time there was over-representation of women with university education and under representation of immigrants from non-English speaking countries [20, 21].

Informed written consent was obtained from all women participating in the Australian Longitudinal study of Women’s Health by the initiating project team with ethical clearance provided by the University of Newcastle (University of Newcastle HREC EC00144 (2004/HE000224). Ethical approval is not required for researchers who are granted access to the de-identified data.

### Sample

**Fig 1:**
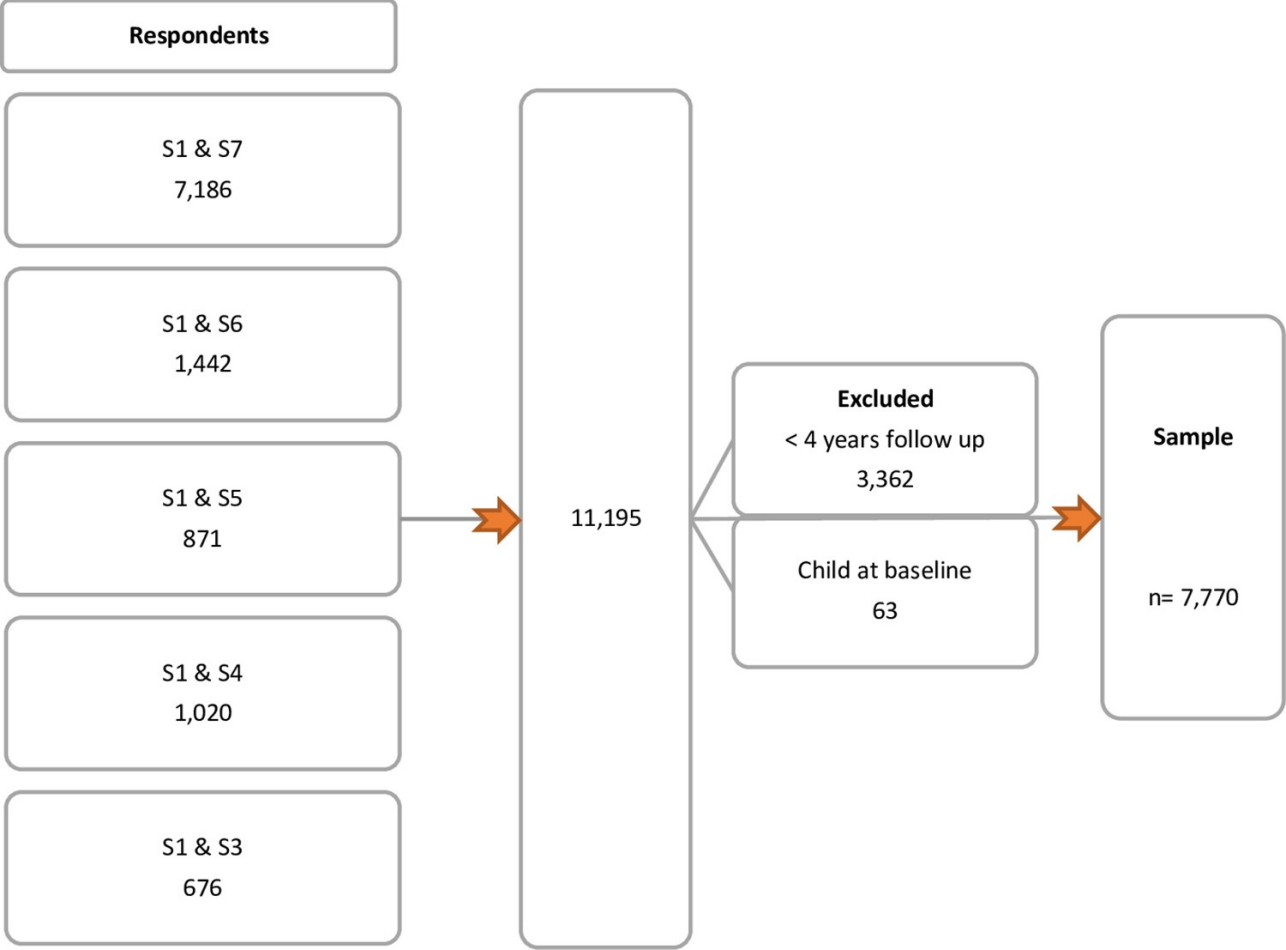
Sample selection.

To select the women whose data would be included in these prospective analyses, we first identified 7,186 women who participated in both the baseline survey (S1) in 1996 and in the seventh survey in 2015 (S7). If women did not participate in S7, we identified women who participated in S1 and S6 (*n* = 1,442) followed by S1 and S5 (*n* = 871), S1 and S4 (*n* = 1,020) and S1 and S3 (*n* = 676). We used data on the reproductive history of these 11,195 women (available from S1 to S6) to calculate the time between each woman’s last birth and their last available survey-data (the follow-up survey). Women with less than 4 years follow-up (*n* = 3,362) and those who already had a child at baseline in 1996, but with no further information about their reproductive history, were excluded (*n* = 63) resulting in an analysis sample of 7,770 women. (see Fig 1).

### Outcome

Health related quality of life, as measured by the Medical Outcomes Study (MOS) Short Form Survey Instrument (SF-36) (Australian standard version) [18], was assessed at baseline (1996) and follow-up which was the latest available survey for each woman. The SF36 includes eight dimensions of physical and mental health: physical functioning (PF, 10 items); role limitations due to physical (RP) or emotional (RE) problems (four items each); bodily pain (BP, two items); general health (GH, five items); vitality (VT, four items); social functioning (SF, two items); and mental health (MH, five items). Responses to items within each dimension were summed and linearly transformed to produce dimension scores ranging from 0 (lowest well-being) to 100 (highest wellbeing) and summary scores for the physical and mental components (abbreviated as PCS and MCS), standardised to population norms and set to have a mean of 50 and a SD of 10 [22]. PCS was based on the subscales PF, RP, BP, and GH and MCS was based on the subscales VT, RE, SF and MH.

### Exposure

The exposures of interest were parity and mode of birth, drawn from questions in surveys 2–6 about reproductive history (Copies of the ALSHW surveys, showing the exact wording of each question, can be obtained from https://www.alswh.org.au/for-data-users/data-documentation/surveys/). Information on parity was categorized as 0, 1, 2, or 3+ births. Multiple births (twins, triplets etc.) counted as only one birth (*n* = 102). Mode of birth was categorized as: spontaneous vaginal birth(s) (VB); vaginal birth(s) with one or more instrumental (VBI); cesarean section(s) (CS); mixed vaginal birth(s) and cesarean section(s), but the last birth by cesarean section (VCS); or mixed vaginal birth(s) and cesarean section(s), but the last birth a vaginal birth (CSV). Women with no reproductive history were categorized as “no births” (NB). Because 77 women did not provide any information about mode of birth, the sample was slightly reduced to 7693 women for this analysis.

### Statistical analyses

Descriptive statistics were used to present sample characteristics at the baseline survey (S1, 1996) according to parity and mode of birth. Continuous variables were reported as means and standard deviations, and categorical variables as frequencies and proportions.

Linear regression models were used to estimate prospective associations between parity and mode of birth with eight subscale and two summary scores of the SF36, assessed after a mean follow-up of 11 years, and differences between SF36 scores at follow up for women in different parity and mode of birth categories. As reference, we used parity 2 (because it is the norm in Australia) and spontaneous vaginal birth(s). In adjusted analyses, we controlled for the following pre-defined potential confounders, measured at baseline (Model 1): age, area of residence, chronic disease, BMI, smoking, ability to walk 100 m, and education. For each analysis of subscales and component summary SF36 scores, we also adjusted for the same SF36 score, measured at baseline. Additionally, we adjusted for the sampling structure i.e., which follow-up survey was used. In Model 2, we added the following variables to Model 1, all measured at follow-up: age, BMI, smoking, ability to walk 100 m, and education. Results were presented as coefficients with their 95% confidence intervals. The coefficients represent mean differences between standardized scores for each parity and birth category and their respective reference group (Parity 2 or spontaneous vaginal birth(s).

In supplementary analyses, we restricted the sample to parous women and adjusted according to Model 2 and further the analysis of parity for mode of birth, and the analysis of mode of birth for parity. Some parous women already had children at baseline (*n* = 751), and the analyses were repeated after excluding these women. STATA16 [23] was used for all analyses.

## Results

In this study of 7,770 Australian women, we observed an average follow-up of 11.0 years (range 4.0–26.1 years) from a woman’s last birth (or baseline for nulliparous women) to her last survey. Baseline characteristics by parity are shown in Table 1, and characteristics by parity at follow up and by mode of birth at baseline and follow-up are presented in (S1–S3 Tables).

**Table 1:** Participant characteristics by parity.

		Nulliparous		Parity = 1		Parity = 2		Parity> = 3	
		mean	SD	mean	SD	mean	SD	mean	SD
Age at baseline		20.53	1.45	20.87	1.50	21.06	1.41	21.16	1.41
Age at follow-up		35.87	4.83	38.04	3.51	38.93	2.75	39.18	2.57
Follow-up duration		15.37	4.53	9.71	4.59	8.47	3.41	7.86	3.08
**Baseline characteristics**		**N**	**%**	**N**	**%**	**N**	**%**	**N**	**%**
Area of residence	Metropolitan	1700	61.33	488	54.22	1364	51.8	650	45.08
	Rural	1001	36.11	371	41.22	1169	44.4	710	49.24
	Remote	71	2.56	41	4.56	100	3.8	82	5.69
Chronic disease	Nil	1464	52.61	413	45.64	1366	51.8	683	47.27
	1–2	1224	43.98	454	50.17	1190	45.13	711	49.2
	3+	95	3.41	38	4.2	81	3.07	51	3.53
Body mass index	<18.5	219	8.67	86	10.72	198	8.35	100	8.13
	18.5–25	1671	66.18	515	64.21	1653	69.75	843	68.54
	25–30	411	16.28	139	17.33	382	16.12	215	17.48
	30–35	151	5.98	46	5.74	105	4.43	48	3.9
	> = 35	73	2.89	16	2	32	1.35	24	1.95
Smoking status	Never	1584	59.24	411	48.01	1357	53.43	715	51.59
	< Weekly	331	12.38	133	15.54	430	16.93	254	18.33
	Weekly	310	11.59	86	10.05	306	12.05	148	10.68
	Daily	449	16.79	226	26.4	447	17.6	269	19.41
Ability to walk 100 m	Not limited	2657	96.51	853	94.78	2529	96.79	1380	96.3
	Limited	96	3.49	47	5.22	84	3.21	53	3.7
Education	Low (school only)	1966	70.97	636	70.67	1723	65.71	975	67.66
	Middle (technical)	470	16.97	176	19.56	530	20.21	269	18.67
	High (university)	334	12.06	88	9.78	369	14.07	197	13.67
Follow up survey	7^th^	1515	54.44	630	69.61	2073	78.61	1155	79.93
	6^th^	287	10.31	117	12.93	313	11.87	174	12.04
	5^th^	235	8.44	68	7.51	130	4.93	68	4.71
	4^th^	396	14.23	66	7.29	86	3.26	41	2.84
	3^rd^	350	12.58	24	2.65	35	1.33	7	0.48

Mean age at follow-up was highest in the parity 3 group, and average follow-up ranged from 7.9 years in the parity 3 group to 15.4 years in nulliparous women. At baseline there were some differences between groups in demographic characteristics. For example, nulliparous women were less likely to live in rural and remote areas, while an increasing trend in the proportion of women residing in rural or remote regions was seen with increasing parity (0–3+). Compared with other parity groups, women who had only one birth were less likely to be highly educated and more likely to have chronic disease, be limited in their ability to walk 100 meters, and to smoke daily.

Results are presented in figures in this manuscript and supporting information provides all results in table format, S4 Table (parity) and S5 Table (mode of birth).

### Parity

**Fig 2:**
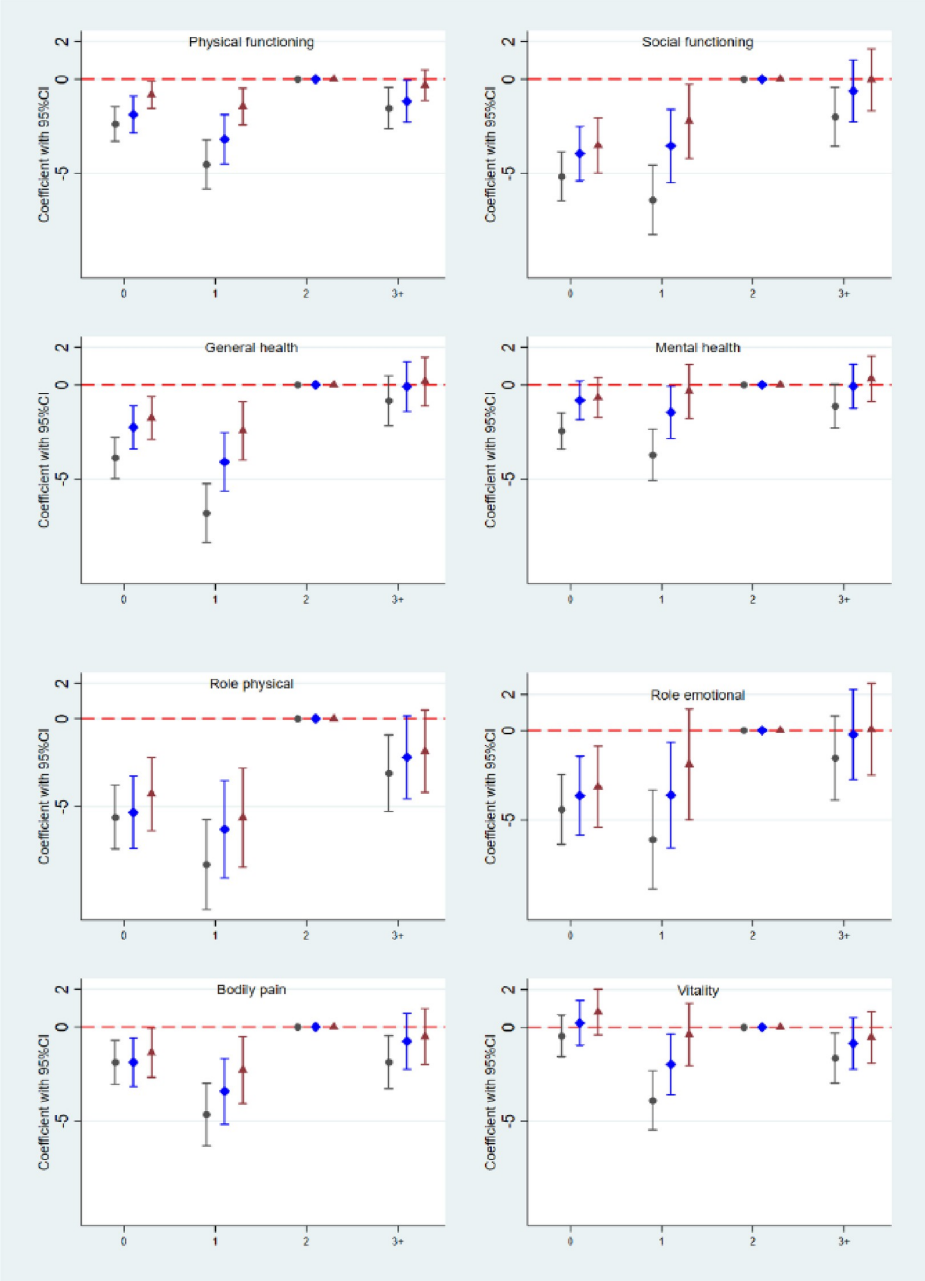
Parity and differences in the physical (left column) and mental (right column) health subscales of the SF36. (0: Parity 0; 1: Parity 1; 2: Parity 2 (reference); 3+: Parity 3 plus. Black circle: crude analysis. Blue diamond: Model 1 with adjustment for sampling structure, and for age, area of residence, chronic disease, BMI, smoking, ability to walk 100 m, education, and specific subscale SF 36 score, all measured at baseline. Red triangle: Model 2: As Model 1 and further adjustment for age, BMI, smoking, ability to walk 100 m, and education, all measured at follow-up.

Results for the SF36 subscale scores are shown by parity in Fig 2.

In the unadjusted models, scores for all four physical health subscales (left hand panel of Fig 2) were lower for women in the parity 0 and parity 1 groups than in the reference group (parity 2), and these differences persisted after adjusting for both baseline confounders (Model 1) and follow-up variables (Model 2). Scores were also lower for parity 3+ for PF, RP and BP, than in the reference group but these differences only remained for PF after adjustment for baseline confounders, and were no longer present after full adjustment.

**Fig 3:**
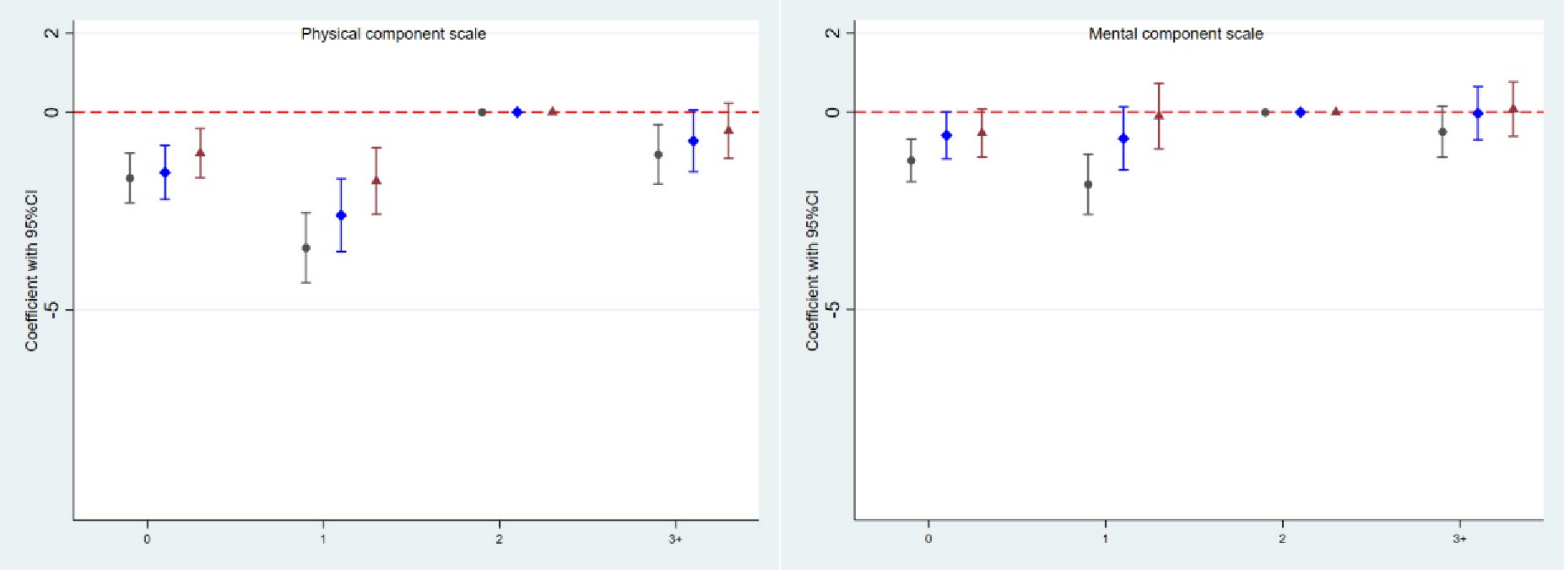
Parity and differences in physical and mental component summary scales. (0: Parity 0; 1: Parity 1; 2: Parity 2 (reference); 3+: Parity 3 plus. Black circle: crude analysis. Blue diamond: Model 1 with adjustment for sampling structure, and for age, area of residence, chronic disease, BMI, smoking, ability to walk 100 m, education, and specific component summary SF36 scores, all measured at baseline. Red triangle: Model 2: As Model 1 and further adjustment for age, BMI, smoking, ability to walk 100 m, and education, all measured at follow-up.

For the mental health subscales (right hand panel), scores were lower in the parity 0 group than in the reference group for SF, MH, and RF and persisted after full adjustment for SF and RE. In parity 1, scores for all four subscales (SF, MH, RE and VT) were lower than in the reference group. These differences remained after adjustment for baseline confounders, but after full adjustment, only persisted for SF. Scores were also lower for SF and VT in parity 3+ than in the reference group in unadjusted models, though not after accounting for baseline confounders. PCS and MCS scores are shown by parity in Fig 3.

Compared with PCS scores in the parity 2 group, PCS scores were markedly lower in women in the parity 0 and parity 1 groups, even after full adjustment. MCS scores were also lower in the parity 0 and 1 groups than in parity 2, but the differences were attenuated after adjustment, especially in the parity 1 group.

### Mode of birth

**Fig 4:**
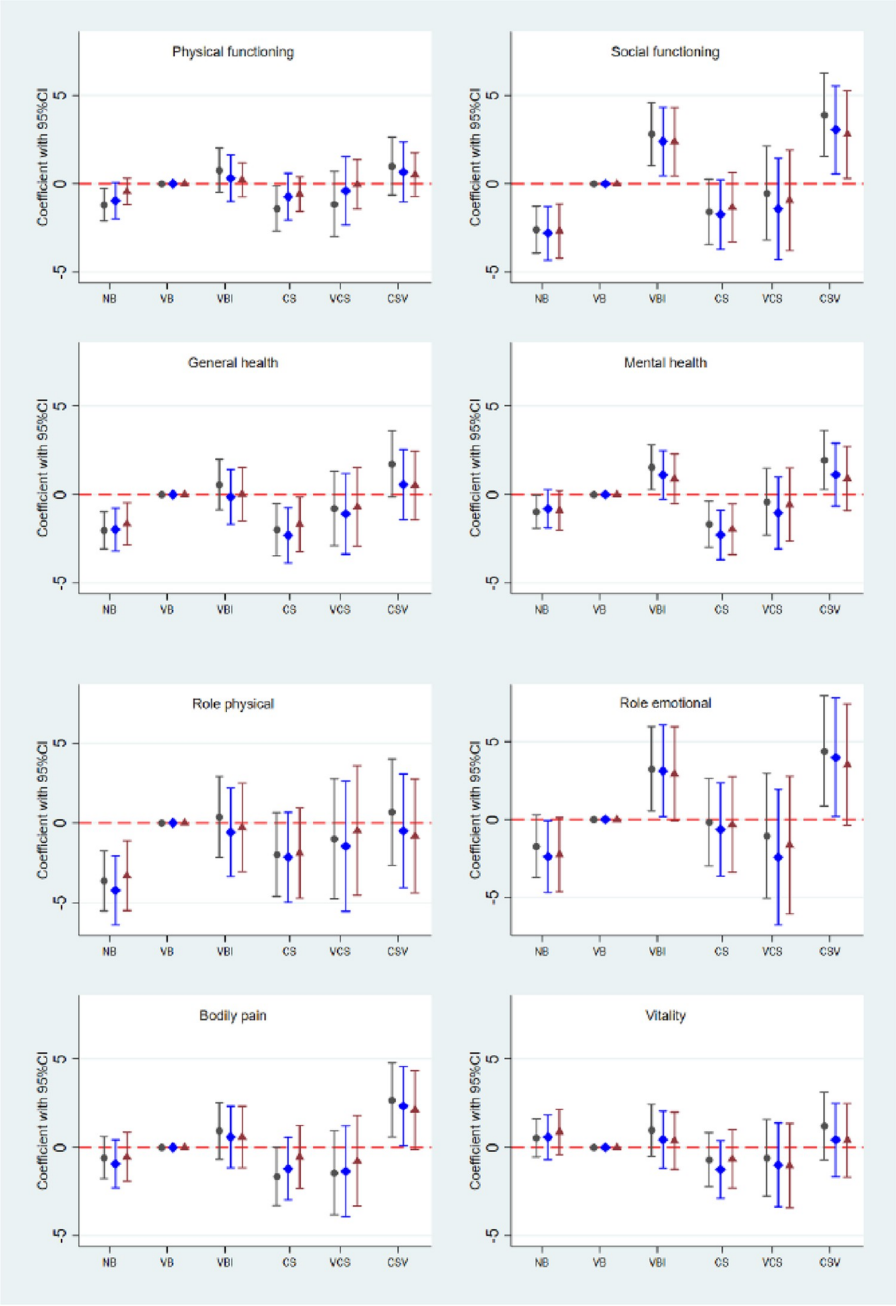
Mode of birth and differences in physical (left column) and mental (right column) health subscales of the SF36. No reproductive history “no births” (NB); spontaneous vaginal birth(s) (VB); vaginal birth(s) with one or more instrumental (VBI); cesarean section(s) (CS); mixed vaginal birth(s) and cesarean section(s), but the last birth by cesarean section (VCS); or mixed vaginal birth(s) and cesarean section(s), but the last birth a vaginal birth (CSV). Reference group is VB. Black circle: crude analysis. Blue diamond: Model 1 with adjustment for sampling structure, and for age, area of residence, chronic disease, BMI, smoking, ability to walk 100 m, education, and specific subscale SF 36 score, all measured at baseline. Red triangle: Model 2: As Model 1 and further adjustment for age, BMI, smoking, ability to walk 100 m, and education, all measured at follow-up.

Results for the SF36 subscale scores are shown by mode of birth in Fig 4.

In the unadjusted models for physical subscales (left hand panel of Fig 4), scores were lower for women with no births for PF, GH and RP than in women with spontaneous vaginal birth(s)(reference group). After adjusting for baseline confounders, these differences persisted for GH and RP only. Scores were also lower for women with only caesarean section(s) than in women with spontaneous vaginal births for PF and GH, with differences remaining for GH after baseline adjustment. Scores for BP were higher in women in the mixed group (including both vaginal birth(s) and caesarean section(s), whose last birth was vaginal than in the reference group. This difference remained after adjustment for baseline variables, but not after full adjustment.

**Fig 5:**
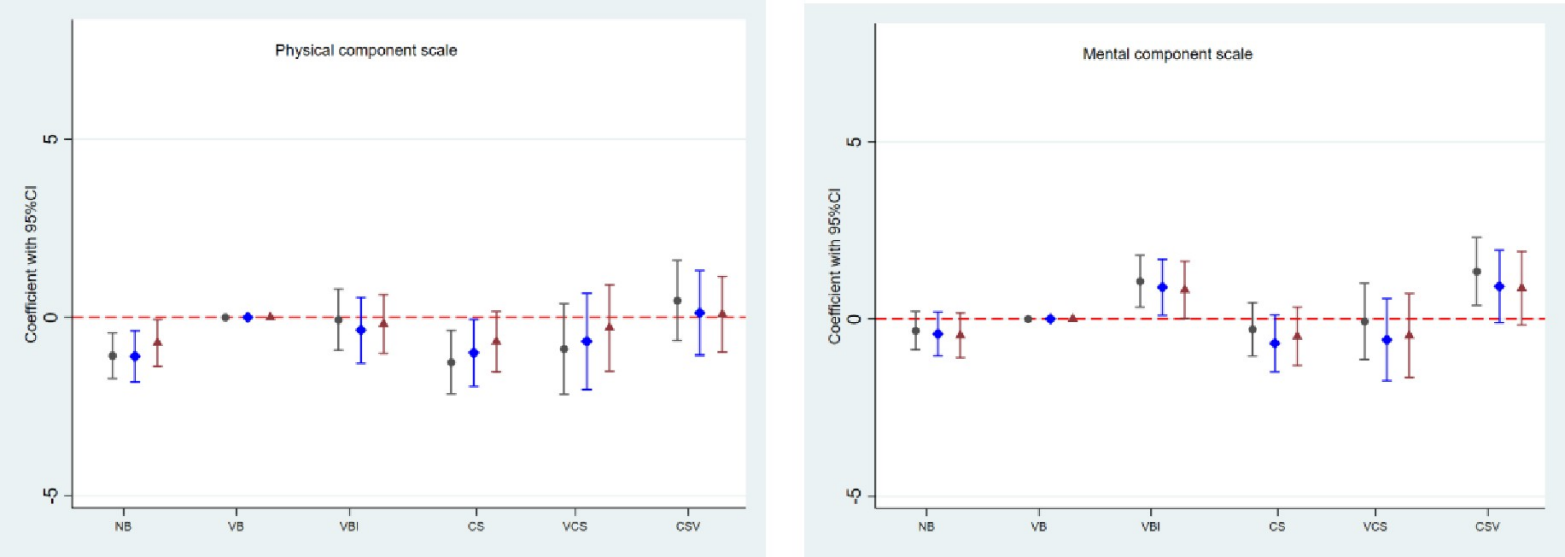
Mode of birth and differences in SF36 component summary scores. No reproductive history “no births” (NB); spontaneous vaginal birth(s) (VB) (reference); vaginal birth(s) with one or more instrumental (VBI); cesarean section(s) (CS); mixed vaginal birth(s) and cesarean section(s), but the last birth by cesarean section (VCS); or mixed vaginal birth(s) and cesarean section(s), but the last birth a vaginal birth (CSV). Reference group is VB. Black circle: crude analysis. Blue diamond: Model 1 with adjustment for sampling structure, and for age, area of residence, chronic disease, BMI, smoking, ability to walk 100 m, education, and specific component summary SF36 scores, all measures at baseline. Red triangle: Model 2: As Model 1 and further adjustment for age, BMI, smoking, ability to walk 100 m, and education, all measured at follow-up.

For the mental health subscales (right hand panel), when compared with women who had spontaneous vaginal birth(s), lower scores for SF were only observed in women with no births; a result that persisted after full adjustment. Scores for SF, MH and RE were higher in women with vaginal birth(s) with one or more instrumental birth(s) than in the reference group. After full adjustment, these differences remained for SF. Finally, scores for SF, MH, and RE were higher in women in the mixed group, whose last birth was vaginal than in the reference group. After confounder adjustment, these differences remained for SF and RE, and after full adjustment, only for SF. Physical and mental component summary scales for each mode of birth are shown in Fig 5.

Compared with women with spontaneous vaginal birth(s), women with no births and women with only caesarean section(s) had lower PCS scores, which remained in both groups after confounder adjustment, but only in the “no birth” group after full adjustment. For MCS, scores were higher for women with vaginal birth(s) with one or more instrumental birth(s) and in women in the mixed group, whose last birth was vaginal than in the reference group. In the latter group, these differences attenuated after adjustment.

In sensitivity analyses restricted to parous women, we adjusted parity for mode of birth and vice versa. This had only minor effects on the observed differences and did not change the findings reported above, except that lower SF in parity 1 women was attenuated and no longer statistically significant. Excluding women with births before baseline did not change the findings.

## Discussion

### Main findings

The aim was to compare QoL over a period of 4–26 years in women with and without children, and in women who experienced different modes of birth. Overall, nulliparous women and those with one child had lower QoL scores than those with two children, especially on the physical health and social functioning scales. Women who experienced birth by caesarean section also had lower scores on the GH and MH subscales of the SF36 than women who had only vaginal birth(s).

### Strengths and limitations

The strengths of this study include the representative sample established for the Australian Longitudinal Study on Women’s Health [24], the inclusion of a nulliparous group, the longitudinal analyses with adjustment for many potential confounding variables, and average follow up period of more than 10 years. We further adjusted for the same factors at follow-up in a separate analysis, but as these factors may be part of the pathway from our exposures to QoL, these results should be interpreted with caution. Without any possibility of randomised controlled trials in this area, observational studies such as this, provide us with the best evidence possible. The limitations include the potential bias due to unmeasured confounders and the potential for selection bias due to attrition from the longitudinal study. As all information was self-reported, we expect some misclassification of mode of birth, but this would most likely be non-differential and thus bias the estimates against the null. A main limitation of all observational studies is the inability to attribute causation between the exposure and outcome variables.

### Interpretation

#### Parity

This is the first study to compare QoL several years after the last birth, in women with different numbers of children. Women who remained nulliparous, and those who had only one birth, had lower scores on all the physical subscales and on the social functioning scale of the SF36. Similar findings have not been previously reported for women of parity 1.

Childlessness may be by choice or involuntary, and both qualitative and quantitative studies have highlighted the psychological distress experienced by women who are involuntarily childless [25–27] or undergoing fertility treatment. Drawing on studies mostly conducted in the clinical infertility treatment setting and using the SF36, a systematic review identified that when compared with comparative data, women with infertility have lower scores on mental health, social functioning, role emotional and emotional behaviour components of the SF 36 [28]. We were not able to distinguish between voluntary and involuntary childlessness.

A Norwegian longitudinal study of wellbeing in a mid-life cohort of individuals also found an association between parental status and “cognitive wellbeing” (satisfaction with life, self-esteem) but not with “affective wellbeing” (happiness, sadness, joy, depression). Childless women reported lower life satisfaction and self-esteem than those with children. Interestingly, the same effects were not found in mid-life men [29].

Our finding that women with no births or only one birth had lower scores on the social functioning subscale may reflect the fact that many social connections are made through parenting roles. Childlessness, especially when it is involuntary, can be associated with feelings of failure and low self-esteem. Women who are childless may therefore experience a greater sense of social isolation and social stigma [35]. Wirtberg, Möller [30] for example found that 20 years after unsuccessful fertility treatment, women were still experiencing social isolation, and for some there was a strong resurgence of loneliness as their peers moved into the grandparenting phase of their lives.

One characteristic of the sociocultural context that influences infertility is pro-natalism

(Parry 2005, Ulrich and Weatherall 2000). While all societies are pro-natalist, some

emphasise the centrality of motherhood to women’s identity more than others

One characteristic of the sociocultural context that influences infertility is pro-natalism

(Parry 2005, Ulrich and Weatherall 2000). While all societies are pro-natalist, some

emphasise the centrality of motherhood to women’s identity more than others

One characteristic of the sociocultural context that influences infertility is pro-natalism

(Parry 2005, Ulrich and Weatherall 2000). While all societies are pro-natalist, some

emphasise the centrality of motherhood to women’s identity more than others.

#### Mode of birth

Mode of birth can have a major impact on women and researchers have called for studies that measure more than physical outcomes [5] and include longer follow up periods [31]. Our study addresses these issues by examining the impact of mode of birth on QoL, with a mean follow up period of more than 10 years. Ours is the only study to date to include a non-parous group of women for comparison.

Most studies of mode of birth and QoL examine differences between women who experienced caesarean section or vaginal birth, with follow up periods for the most part of 6 months or less [6–11] and occasionally up to 12 months [12, 13]. Results of these studies vary considerably, reflecting the heterogeneity in population, duration of follow up, and in the instrument used to assess QoL. Two systematic reviews with meta-analysis by the same lead author in the same year reported conflicting results and are of dubious quality; one showed no significant differences between groups [32] and the other showed differences in most dimensions of QoL [33]. A disproportionate number of studies on this topic originate in Iran [6–8, 12, 34–36] with relatively few studies in European or Western settings [9–11, 13].

An English study of more than 2000 women examined QoL using the EuroQol Five Dimensions (EQ-5D) instrument with 12 months follow up. The researchers found that a significantly higher proportion of women who gave birth by caesarean section reported pain at 12 months postpartum, compared with vaginal birth [13]. Our study identified no differences in bodily pain between these groups, which may reflect the longer duration of follow up. A Swedish study with 5-year follow-up examined differences in QoL and mode of delivery using the SWED-QUAL instrument. The researchers reported lower health related QoL in women who underwent emergency caesarean section, than in those who had a vaginal birth, instrumental vaginal birth or caesarean section on maternal request [18]. Our study was not able to determine indications for caesarean section, but we found that women who experienced caesarean section had lower scores in the General and Mental Health subscales of the SF36 than women who experienced vaginal birth.

In our study the “no birth” group had lower scores on the physical but not the mental component summary scores which may suggest that the “no birth” status was due to poorer physical health. As in the results of our analyses by parity, the “no birth” group had significantly lower scores on the social functioning subscale. This contrasts with the findings for the mixed vaginal/assisted birth and caesarean section with last birth vaginal groups, which both had higher scores than women with spontaneous vaginal births. This may reflect a sense of achievement and self-esteem for these groups which carried through to their social spheres of life. It may also reflect an underlying stronger mental and physical health that may also increase the likelihood of opting for a vaginal birth after caesarean section.

### Implications

Increasing rates of caesarean section has caused concern as the longer-term consequences for women’s health are not well understood. The minimum important difference (MID) for the SF36 subscales and component summary scores have not been defined for this population however, differences of 2 and 3 for physical and mental component summary respectively and 2–4 on subscales have been recommended [37]. In our study comparisons between the caesarean section and vaginal birth groups did not reach this threshold suggesting that caesarean section has little impact on women’s health in the longer term. Important differences were found in parity, particularly in women with one child, compared to those with two. While childlessness is understood as a factor potentially associated with physical, social, emotional or mental health issues in women, having one child has not. This study provides health care professionals with awareness of potential impacts on women’s wellbeing from having fewer children that is the norm in Australia.

## Conclusion

Our study makes a unique contribution to the body of evidence examining long term effects of parity and mode of birth on QoL. We demonstrate that both women with no births and only one birth have poorer scores on all four physical health and on the social functioning subscales of the SF36. QoL in women with only one birth has previously been overlooked in research and our study highlights its significance and potential for future research. The long-term effects of caesarean section and associations with lower scores on general and mental health are worthy of further study, as are the higher scores on emotional and social functioning for women with a history of caesarean section, but where the last birth was vaginal.

## Supporting information

S1 Table Participant characteristics at baseline by mode of birth. (DOCX)

S2 Table Participant characteristics at follow up by parity. (DOCX)

S3 Table Participant characteristics at follow up by mode of birth. (DOCX)

S4 Table SF36 subscale and component summary coefficients and parity. (DOCX)

S5 Table SF36 subscale and component summary coefficients and mode of birth. (DOCX)
