# Supplementary material for: Parity and mode of birth and their relationships with quality of life: A longitudinal study
Source: PLoS One. 2022 Sep 9;17(9):e0273366. doi: 10.1371/journal.pone.0273366 (PMC9462673; doi:10.1371/journal.pone.0273366)
Supplement: S1 Table — (DOCX) [file pone.0273366.s001.docx]

**S1 Table. Participant characteristics at baseline by mode of birth**

|  |  | **No birth** | | **VB** | | **VBI** | | **CS** | | **VCS** | | **CSV** | |
| --- | --- | --- | --- | --- | --- | --- | --- | --- | --- | --- | --- | --- | --- |
|  |  | Mean | SD | Mean | SD | Mean | SD | Mean | SD | Mean | SD | Mean | SD |
| Age at baseline |  | 20.53 | 1.45 | 21.01 | 1.42 | 21.09 | 1.45 | 21.03 | 1.45 | 21.18 | 1.40 | 21.09 | 1.39 |
| Age at outcome |  | 35.87 | 4.83 | 38.75 | 2.83 | 39.13 | 2.53 | 38.96 | 2.53 | 39.14 | 2.54 | 39.69 | 1.85 |
| Follow-up duration |  | 15.34 | 4.53 | 9.07 | 3.88 | 8.31 | 3.46 | 8.35 | 3.51 | 8.23 | 3.07 | 7.11 | 2.67 |
|  |  |  |  |  |  |  |  |  |  |  |  |  |  |
| **Baseline characteristics** | | **N** | **%** | **N** | **%** | **N** | **%** | **N** | **%** | **N** | **%** | **N** | **%** |
| Area of residence | Metropolitan | 1700 | 61.33 | 1054 | 47.46 | 523 | 54.14 | 478 | 54.2 | 147 | 41.53 | 271 | 56.93 |
|  | Rural | 1001 | 36.11 | 1055 | 47.5 | 401 | 41.51 | 372 | 42.18 | 189 | 53.39 | 189 | 39.71 |
|  | Remote | 71 | 2.56 | 112 | 5.04 | 42 | 4.35 | 32 | 3.63 | 18 | 5.08 | 16 | 3.36 |
| Chronic disease | Nil | 1464 | 52.61 | 1111 | 49.87 | 473 | 48.81 | 464 | 52.61 | 160 | 45.2 | 226 | 47.38 |
|  | 1-2 | 1224 | 43.98 | 1039 | 46.63 | 465 | 47.99 | 390 | 44.22 | 180 | 50.85 | 237 | 49.69 |
|  | 3+ | 95 | 3.41 | 78 | 3.5 | 31 | 3.2 | 28 | 3.17 | 14 | 3.95 | 14 | 2.94 |
| BMI | <18.5 | 219 | 8.67 | 173 | 8.91 | 83 | 9.67 | 67 | 8.31 | 18 | 5.81 | 38 | 8.68 |
|  | 18.5-25 | 1671 | 66.18 | 1358 | 69.93 | 600 | 69.93 | 528 | 65.51 | 201 | 64.84 | 294 | 67.12 |
|  | 25-30 | 411 | 16.28 | 300 | 15.45 | 135 | 15.73 | 135 | 16.75 | 71 | 22.9 | 84 | 19.18 |
|  | 30-35 | 151 | 5.98 | 87 | 4.48 | 31 | 3.61 | 52 | 6.45 | 15 | 4.84 | 12 | 2.74 |
|  | >=35 | 73 | 2.89 | 24 | 1.24 | 9 | 1.05 | 24 | 2.98 | 5 | 1.61 | 10 | 2.28 |
| Smoking status | Never | 1584 | 59.24 | 1097 | 50.98 | 500 | 54.41 | 435 | 51.6 | 173 | 50.58 | 252 | 55.51 |
|  | < Weekly | 331 | 12.38 | 369 | 17.15 | 169 | 18.39 | 120 | 14.23 | 68 | 19.88 | 74 | 16.3 |
|  | Weekly | 310 | 11.59 | 248 | 11.52 | 90 | 9.79 | 102 | 12.1 | 45 | 13.16 | 49 | 10.79 |
|  | Daily | 449 | 16.79 | 438 | 20.35 | 160 | 17.41 | 186 | 22.06 | 56 | 16.37 | 79 | 17.4 |
| Ability to walk 100 m | Not limited | 2657 | 96.51 | 2125 | 96.15 | 919 | 95.63 | 842 | 96.34 | 342 | 97.16 | 468 | 98.73 |
|  | Limited | 96 | 3.49 | 85 | 3.85 | 42 | 4.37 | 32 | 3.66 | 10 | 2.84 | 6 | 1.27 |
| Education | Low | 1966 | 70.97 | 1518 | 68.32 | 645 | 67.05 | 574 | 65.38 | 226 | 64.39 | 303 | 63.92 |
|  | Middle | 470 | 16.97 | 427 | 19.22 | 191 | 19.85 | 185 | 21.07 | 74 | 21.08 | 92 | 19.41 |
|  | High | 334 | 12.06 | 277 | 12.47 | 126 | 13.1 | 119 | 13.55 | 51 | 14.53 | 79 | 16.67 |
| Follow-up survey | 7^th^ | 1515 | 54.44 | 1685 | 75.63 | 771 | 79.57 | 686 | 77.78 | 276 | 77.97 | 433 | 90.78 |
|  | 6^th^ | 287 | 10.31 | 282 | 12.66 | 121 | 12.49 | 120 | 13.61 | 46 | 12.99 | 33 | 6.92 |
|  | 5^th^ | 235 | 8.44 | 144 | 6.46 | 47 | 4.85 | 44 | 4.99 | 22 | 6.21 | 8 | 1.68 |
|  | 4^th^ | 396 | 14.23 | 117 | 5.25 | 30 | 3.1 | 32 | 3.63 | 10 | 2.82 | 3 | 0.63 |
|  | 3^rd^ | 350 | 12.58 | 0 | 0 | 0 | 0 | 0 | 0 | 0 | 0 | 0 | 0 |
| Spontaneous vaginal birth(s) (VB); vaginal birth(s) with one or more instrumental (VBI); cesarean section(s) (CS); mixed vaginal birth(s) and cesarean section(s), but the last birth by cesarean section (VCS); or mixed vaginal birth(s) and cesarean section(s), but the last birth a vaginal birth (CSV). | | | | | | | | | | | | | |
