# Supplementary material for: Parity and mode of birth and their relationships with quality of life: A longitudinal study
Source: PLoS One. 2022 Sep 9;17(9):e0273366. doi: 10.1371/journal.pone.0273366 (PMC9462673; doi:10.1371/journal.pone.0273366)
Supplement: S2 Table — (DOCX) [file pone.0273366.s002.docx]

**S2. Participant characteristics at follow up by parity**

|  |  | **Nulliparous** | | | **Parity=1** | | **Parity=2** | | **Parity>=3** | |
| --- | --- | --- | --- | --- | --- | --- | --- | --- | --- | --- |
|  |  | Mean | SD | Mean | | SD | Mean | SD | Mean | SD |
| Age at baseline |  | 20.5344 | 1.4482 | | 20.8737 | 1.4955 | 21.0578 | 1.4074 | 21.159 | 1.4125 |
| Age at follow-up |  | 35.8711 | 4.8315 | | 38.038 | 3.5138 | 38.9274 | 2.7546 | 39.182 | 2.5718 |
| Follow-up duration |  | 15.3366 | 4.534 | | 9.7096 | 4.5856 | 8.4671 | 3.406 | 7.8609 | 3.0794 |
|  |  |  |  | |  |  |  |  |  |  |
| **Baseline characteristics** | | **N** | **%** | | **N** | **%** | **N** | **%** | **N** | **%** |
| BMI | <18.5 | 78 | 3 | | 20 | 2.38 | 44 | 1.74 | 24 | 1.75 |
|  | 18.5-25 | 1197 | 46.06 | | 311 | 36.98 | 1128 | 44.62 | 587 | 42.75 |
|  | 25-30 | 618 | 23.78 | | 224 | 26.64 | 707 | 27.97 | 388 | 28.26 |
|  | 30-35 | 351 | 13.51 | | 163 | 19.38 | 392 | 15.51 | 211 | 15.37 |
|  | >=35 | 355 | 13.66 | | 123 | 14.63 | 257 | 10.17 | 163 | 11.87 |
| Smoking status | Never | 2208 | 81.54 | | 672 | 77.51 | 2234 | 86.99 | 1182 | 84.25 |
|  | < Weekly | 120 | 4.43 | | 30 | 3.46 | 53 | 2.06 | 23 | 1.64 |
|  | Weekly | 47 | 1.74 | | 13 | 1.5 | 22 | 0.86 | 16 | 1.14 |
|  | Daily | 333 | 12.3 | | 152 | 17.53 | 259 | 10.09 | 182 | 12.97 |
| Ability to walk 100 m | Not limited | 2603 | 94.48 | | 842 | 93.45 | 2539 | 96.8 | 1374 | 95.55 |
|  | Limited | 152 | 5.52 | | 59 | 6.55 | 84 | 3.2 | 64 | 4.45 |
| Education | Low | 446 | 16.79 | | 225 | 26.95 | 575 | 23.07 | 361 | 26.35 |
|  | Middle | 720 | 27.11 | | 292 | 34.97 | 797 | 31.98 | 450 | 32.85 |
|  | High | 1490 | 56.1 | | 318 | 38.08 | 1120 | 44.94 | 559 | 40.8 |
| Mode of birth | No birth | 2783 | 100 | | 0 | 0 | 0 | 0 | 0 | 0 |
|  | VB | 0 | 0 | | 374 | 41.33 | 1129 | 42.81 | 725 | 50.17 |
|  | VBI | 0 | 0 | | 150 | 16.57 | 541 | 20.52 | 278 | 19.24 |
|  | CS | 0 | 0 | | 351 | 38.78 | 452 | 17.14 | 79 | 5.47 |
|  | VCS | 0 | 0 | | 0 | 0 | 214 | 8.12 | 140 | 9.69 |
|  | CSV | 0 | 0 | | 0 | 0 | 262 | 9.94 | 215 | 14.88 |
|  | Missing | 0 | 0 | | 30 | 3.31 | 39 | 1.48 | 8 | 0.55 |
|  |  |  |  | |  |  |  |  |  |  |
