# Supplementary material for: Parity and mode of birth and their relationships with quality of life: A longitudinal study
Source: PLoS One. 2022 Sep 9;17(9):e0273366. doi: 10.1371/journal.pone.0273366 (PMC9462673; doi:10.1371/journal.pone.0273366)
Supplement: S3 Table — (DOCX) [file pone.0273366.s003.docx]

**S3. Participant characteristics at follow up by mode of birth**

|  |  | **No birth** | | **VB** | | **VBI** | | **CS** | | **VCS** | | **CSV** | |
| --- | --- | --- | --- | --- | --- | --- | --- | --- | --- | --- | --- | --- | --- |
|  |  | Mean | SD | Mean | SD | Mean | SD | Mean | SD | Mean | SD | Mean | SD |
| Age at baseline |  | 20.53 | 1.45 | 21.01 | 1.42 | 21.09 | 1.45 | 21.03 | 1.45 | 21.18 | 1.40 | 21.09 | 1.39 |
| Age at outcome |  | 35.87 | 4.83 | 38.75 | 2.83 | 39.13 | 2.53 | 38.96 | 2.53 | 39.14 | 2.54 | 39.69 | 1.85 |
| Follow-up duration |  | 15.34 | 4.53 | 9.07 | 3.88 | 8.31 | 3.46 | 8.35 | 3.51 | 8.23 | 3.07 | 7.11 | 2.67 |
|  |  |  |  |  |  |  |  |  |  |  |  |  |  |
| **Baseline characteristics** | | **N** | **%** | **N** | **%** | **N** | **%** | **N** | **%** | **N** | **%** | **N** | **%** |
| BMI | <18.5 | 78 | 3 | 48 | 2.27 | 16 | 1.73 | 12 | 1.44 | 4 | 1.17 | 6 | 1.29 |
|  | 18.5-25 | 1197 | 46.06 | 942 | 44.58 | 414 | 44.81 | 308 | 36.89 | 134 | 39.07 | 199 | 42.89 |
|  | 25-30 | 618 | 23.78 | 584 | 27.64 | 265 | 28.68 | 245 | 29.34 | 86 | 25.07 | 126 | 27.16 |
|  | 30-35 | 351 | 13.51 | 327 | 15.48 | 126 | 13.64 | 152 | 18.2 | 66 | 19.24 | 82 | 17.67 |
|  | >=35 | 355 | 13.66 | 212 | 10.03 | 103 | 11.15 | 118 | 14.13 | 53 | 15.45 | 51 | 10.99 |
| Smoking status | Never | 2208 | 81.54 | 1806 | 83.5 | 820 | 87.7 | 696 | 81.98 | 290 | 83.81 | 426 | 91.03 |
|  | < Weekly | 120 | 4.43 | 52 | 2.4 | 21 | 2.25 | 16 | 1.88 | 8 | 2.31 | 3 | 0.64 |
|  | Weekly | 47 | 1.74 | 29 | 1.34 | 10 | 1.07 | 5 | 0.59 | 3 | 0.87 | 3 | 0.64 |
|  | Daily | 333 | 12.3 | 276 | 12.76 | 84 | 8.98 | 132 | 15.55 | 45 | 13.01 | 36 | 7.69 |
| Ability to walk 100 m | Not limited | 2603 | 94.48 | 2124 | 95.68 | 926 | 96.16 | 838 | 95.66 | 337 | 95.47 | 464 | 97.68 |
|  | Limited | 152 | 5.52 | 96 | 4.32 | 37 | 3.84 | 38 | 4.34 | 16 | 4.53 | 11 | 2.32 |
| Education | Low | 446 | 16.79 | 538 | 25.47 | 217 | 23.93 | 189 | 23.13 | 85 | 25.37 | 89 | 19.69 |
|  | Middle | 720 | 27.11 | 718 | 34 | 277 | 30.54 | 278 | 34.03 | 122 | 36.42 | 120 | 26.55 |
|  | High | 1490 | 56.1 | 856 | 40.53 | 413 | 45.53 | 350 | 42.84 | 128 | 38.21 | 243 | 53.76 |
| Parity | 0 | 2783 | 100 | 0 | 0 | 0 | 0 | 0 | 0 | 0 | 0 | 0 | 0 |
|  | 1 | 0 | 0 | 374 | 16.79 | 150 | 15.48 | 351 | 39.8 | 0 | 0 | 0 | 0 |
|  | 2 | 0 | 0 | 1129 | 50.67 | 541 | 55.83 | 452 | 51.25 | 214 | 60.45 | 262 | 54.93 |
|  | 3+ | 0 | 0 | 725 | 32.54 | 278 | 28.69 | 79 | 8.96 | 140 | 39.55 | 215 | 45.07 |
| Spontaneous vaginal birth(s) (VB); vaginal birth(s) with one or more instrumental (VBI); cesarean section(s) (CS); mixed vaginal birth(s) and cesarean section(s), but the last birth by cesarean section (VCS); or mixed vaginal birth(s) and cesarean section(s), but the last birth a vaginal birth (CSV). | | | | | | | | | | | | | |
