# Supplementary material for: Parity and mode of birth and their relationships with quality of life: A longitudinal study
Source: PLoS One. 2022 Sep 9;17(9):e0273366. doi: 10.1371/journal.pone.0273366 (PMC9462673; doi:10.1371/journal.pone.0273366)
Supplement: S4 Table — (DOCX) [file pone.0273366.s004.docx]

**S4 Table: SF36 subscale and component summary coefficients and parity**

|  | **Parity** | **0** | **1** | **2** | **3** |
| --- | --- | --- | --- | --- | --- |
| **SF36** | **Model** | **Coeff (low-high)** | **Coeff (low-high)** | **Ref** | **Coeff (low-high)** |
| Physical functioning | Crude | -2.38 (-3.29, -1.46) | -4.52 (-5.82, -3.22) |  | -1.53 (-2.64, -0.43) |
|  | Model 1 | -1.88 (-2.86, -0.91) | -3.19 (-4.50, -1.88) |  | -1.18 (-2.29, -0.06) |
|  | Model 2 | -0.83 (-1.55, -0.12) | -1.45 (-2.42, -0.48) |  | -0.32 (-1.13, 0.49) |
|  | Supp |  | -1.32 (-2.32, -0.32) |  | -0.39 (-1.20, 0.42) |
| Role physical | Crude | -5.60 (-7.41, -3.79) | -8.29 (-10.85, -5.72) |  | -3.10 (-5.28, -0.92) |
|  | Model 1 | -5.33 (-7.39, -3.27) | -6.30 (-9.06, -3.53) |  | -2.20 (-4.55, 0.15) |
|  | Model 2 | -4.28 (-6.36, -2.20) | -5.62 (-8.44, -2.80) |  | -1.86 (-4.20, 0.49) |
|  | Supp |  | -5.07 (-7.95, -2.19) |  | -1.93 (-4.26, 0.39) |
| Bodily pain | Crude | -1.88 (-3.05, -0.71) | -4.65 (-6.31, -3.00) |  | -1.87 (-3.28, -0.47) |
|  | Model 1 | -1.88 (-3.18, -0.59) | -3.43 (-5.17, -1.68) |  | -0.77 (-2.25, 0.72) |
|  | Model 2 | -1.38 (-2.69, -0.07) | -2.29 (-4.06, -0.51) |  | -0.52 (-1.99, 0.95) |
|  | Supp |  | -2.11 (-3.95, -0.26) |  | -0.70 (-2.19, 0.79) |
| General health | Crude | -3.88 (-4.97, -2.78) | -6.81 (-8.36, -5.25) |  | -0.84 (-2.16, 0.48) |
|  | Model 1 | -2.25 (-3.40, -1.10) | -4.09 (-5.65, -2.54) |  | -0.09 (-1.41, 1.22) |
|  | Model 2 | -1.76 (-2.90, -0.62) | -2.44 (-3.99, -0.89) |  | 0.18 (-1.11, 1.46) |
|  | Supp |  | -2.40 (-4.01, -0.80) |  | -0.05 (-1.34, 1.24) |
| Vitality | Crude | -0.47 (-1.57, 0.64) | -3.89 (-5.46, -2.33) |  | -1.65 (-2.98, -0.32) |
|  | Model 1 | 0.23 (-0.97, 1.42) | -1.98 (-3.59, -0.37) |  | -0.86 (-2.23, 0.51) |
|  | Model 2 | 0.80 (-0.41, 2.02) | -0.40 (-2.05, 1.26) |  | -0.54 (-1.92, 0.83) |
|  | Supp |  | -0.16 (-1.88, 1.56) |  | -0.75 (-2.14, 0.64) |
| Social functioning | Crude | -5.17 (-6.47, -3.86) | -6.41 (-8.26, -4.56) |  | -1.99 (-3.56, -0.42) |
|  | Model 1 | -3.95 (-5.39, -2.51) | -3.55 (-5.49, -1.61) |  | -0.63 (-2.28, 1.02) |
|  | Model 2 | -3.52 (-4.98, -2.07) | -2.23 (-4.21, -0.26) |  | -0.03 (-1.67, 1.61) |
|  | Supp |  | -1.60 (-3.61, 0.41) |  | -0.22 (-1.84, 1.40) |
| Role emotional | Crude | -4.41 (-6.37, -2.46) | -6.09 (-8.86, -3.33) |  | -1.55 (-3.90, 0.80) |
|  | Model 1 | -3.65 (-5.85, -1.45) | -3.62 (-6.57, -0.66) |  | -0.23 (-2.74, 2.29) |
|  | Model 2 | -3.14 (-5.41, -0.87) | -1.90 (-4.98, 1.18) |  | 0.06 (-2.50, 2.62) |
|  | Supp |  | -1.27 (-4.41, 1.88) |  | -0.04 (-2.58, 2.50) |
| Mental health | Crude | -2.45 (-3.41, -1.49) | -3.71 (-5.08, -2.35) |  | -1.13 (-2.29, 0.03) |
|  | Model 1 | -0.82 (-1.85, 0.21) | -1.45 (-2.84, -0.07) |  | -0.08 (-1.25, 1.10) |
|  | Model 2 | -0.68 (-1.73, 0.38) | -0.35 (-1.79, 1.09) |  | 0.32 (-0.87, 1.52) |
|  | Supp |  | 0.26 (-1.21, 1.72) |  | 0.08 (-1.10, 1.26) |
| PCS | Crude | -1.67 (-2.29, -1.04) | -3.43 (-4.32, -2.54) |  | -1.07 (-1.82, -0.31) |
|  | Model 1 | -1.53 (-2.21, -0.84) | -2.61 (-3.53, -1.69) |  | -0.73 (-1.51, 0.05) |
|  | Model 2 | -1.03 (-1.65, -0.42) | -1.74 (-2.58, -0.90) |  | -0.47 (-1.17, 0.22) |
|  | Supp |  | -1.67 (-2.54, -0.79) |  | -0.56 (-1.26, 0.15) |
| MCS | Crude | -1.22 (-1.75, -0.68) | -1.82 (-2.58, -1.06) |  | -0.49 (-1.14, 0.15) |
|  | Model 1 | -0.58 (-1.17, 0.01) | -0.66 (-1.46, 0.13) |  | -0.03 (-0.70, 0.65) |
|  | Model 2 | -0.53 (-1.13, 0.08) | -0.09 (-0.92, 0.73) |  | 0.08 (-0.60, 0.77) |
|  | Supp |  | 0.16 (-0.68, 1.01) |  | -0.02 (-0.70, 0.66) |
