# Supplementary material for: Parity and mode of birth and their relationships with quality of life: A longitudinal study
Source: PLoS One. 2022 Sep 9;17(9):e0273366. doi: 10.1371/journal.pone.0273366 (PMC9462673; doi:10.1371/journal.pone.0273366)
Supplement: S5 Table — (DOCX) [file pone.0273366.s005.docx]

**S5 Table: SF36 subscale and component summary coefficients and mode of birth**

|  |  | **No births** | **Vaginal** | **Vaginal/**  **Instrumental** | **Caesarean Only** | **Mixed last Caesarean** | **Mixed last Vaginal** |
| --- | --- | --- | --- | --- | --- | --- | --- |
| **SF36** | **Model** | **Coeff (low-high)** | **Ref** | **Coeff (low-high)** | **Coeff (low-high)** | **Coeff (low-high)** | **Coeff (low-high)** |
| Physical functioning | Crude | -1.19 (-2.12, -0.27) |  | 0.76 (-0.49, 2.01) | -1.40 (-2.69, -0.11) | -1.15 (-3.00, 0.70) | 0.99 (-0.64, 2.62) |
|  | Model 1 | -0.97 (-1.99, 0.05) |  | 0.31 (-1.00, 1.62) | -0.74 (-2.07, 0.59) | -0.41 (-2.35, 1.53) | 0.66 (-1.03, 2.35) |
|  | Model 2 | -0.44 (-1.19, 0.31) |  | 0.22 (-0.74, 1.17) | -0.59 (-1.57, 0.39) | -0.03 (-1.44, 1.37) | 0.51 (-0.72, 1.74) |
|  | Supp |  |  | 0.11 (-0.83, 1.05) | -0.31 (-1.30, 0.69) | -0.21 (-1.60, 1.18) | 0.39 (-0.83, 1.61) |
| Role physical | Crude | 0.39 (-0.83, 1.61) |  | 0.37 (-2.17, 2.91) | -1.99 (-4.61, 0.64) | -1.01 (-4.77, 2.76) | 0.68 (-2.65, 4.00) |
|  | Model 1 | -4.23 (-6.39, -2.07) |  | -0.58 (-3.35, 2.18) | -2.15 (-4.95, 0.65) | -1.45 (-5.53, 2.62) | -0.50 (-4.06, 3.06) |
|  | Model 2 | -3.31 (-5.49, -1.13) |  | -0.29 (-3.06, 2.48) | -1.89 (-4.72, 0.94) | -0.48 (-4.54, 3.59) | -0.82 (-4.38, 2.74) |
|  | Supp |  |  | -0.48 (-3.19, 2.23) | -1.05 (-3.92, 1.81) | -1.23 (-5.22, 2.77) | -1.37 (-4.89, 2.15) |
| Bodily pain | Crude | -0.58 (-1.77, 0.60) |  | 0.94 (-0.66, 2.54) | -1.65 (-3.30, 0.01) | -1.44 (-3.82, 0.94) | 2.68 (0.57, 4.78) |
|  | Model 1 | -0.93 (-2.29, 0.43) |  | 0.58 (-1.16, 2.32) | -1.21 (-2.97, 0.56) | -1.36 (-3.93, 1.21) | 2.34 (0.09, 4.59) |
|  | Model 2 | -0.54 (-1.91, 0.83) |  | 0.58 (-1.16, 2.32) | -0.54 (-2.32, 1.23) | -0.77 (-3.33, 1.78) | 2.12 (-0.11, 4.36) |
|  | Supp |  |  | 0.54 (-1.20, 2.27) | -0.16 (-2.00, 1.68) | -1.15 (-3.71, 1.41) | 1.93 (-0.32, 4.19) |
| General health | Crude | -2.02 (-3.08, -0.97) |  | 0.56 (-0.87, 1.99) | -1.98 (-3.46, -0.51) | -0.78 (-2.89, 1.33) | 1.74 (-0.13, 3.60) |
|  | Model 1 | -1.98 (-3.19, -0.77) |  | -0.13 (-1.68, 1.41) | -2.31 (-3.88, -0.74) | -1.09 (-3.37, 1.18) | 0.56 (-1.42, 2.55) |
|  | Model 2 | -1.65 (-2.85, -0.46) |  | 0.02 (-1.50, 1.54) | -1.68 (-3.23, -0.13) | -0.70 (-2.92, 1.53) | 0.52 (-1.43, 2.46) |
|  | Supp |  |  | -0.10 (-1.61, 1.40) | -1.17 (-2.76, 0.42) | -1.08 (-3.30, 1.13) | 0.14 (-1.81, 2.09) |
| Vitality | Crude | 0.54 (-0.54, 1.62) |  | 0.97 (-0.50, 2.43) | -0.70 (-2.21, 0.82) | -0.59 (-2.76, 1.58) | 1.22 (-0.70, 3.14) |
|  | Model 1 | 0.57 (-0.68, 1.83) |  | 0.43 (-1.18, 2.04) | -1.25 (-2.88, 0.38) | -0.99 (-3.37, 1.38) | 0.42 (-1.66, 2.49) |
|  | Model 2 | 0.87 (-0.40, 2.15) |  | 0.37 (-1.25, 1.99) | -0.65 (-2.30, 1.01) | -1.03 (-3.42, 1.35) | 0.40 (-1.68, 2.48) |
|  | Supp |  |  | 0.37 (-1.24, 1.99) | -0.71 (-2.42, 0.99) | -1.12 (-3.51, 1.27) | 0.61 (-1.49, 2.70) |
| Social functioning | Crude | -2.60 (-3.92, -1.28) |  | 2.80 (1.01, 4.59) | -1.59 (-3.44, 0.26) | -0.53 (-3.19, 2.12) | 3.89 (1.55, 6.24) |
|  | Model 1 | -2.81 (-4.32, -1.29) |  | 2.39 (0.45, 4.32) | -1.74 (-3.70, 0.22) | -1.42 (-4.28, 1.43) | 3.05 (0.56, 5.54) |
|  | Model 2 | -2.69 (-4.21, -1.16) |  | 2.38 (0.44, 4.31) | -1.34 (-3.31, 0.64) | -0.94 (-3.78, 1.90) | 2.79 (0.30, 5.27) |
|  | Supp |  |  | 2.29 (0.40, 4.17) | -0.81 (-2.81, 1.19) | -1.17 (-3.96, 1.62) | 2.62 (0.17, 5.07) |
| Role emotional | Crude | -1.72 (-3.72, 0.29) |  | 3.25 (0.54, 5.96) | -0.17 (-2.97, 2.63) | -1.05 (-5.07, 2.96) | 4.41 (0.85, 7.96) |
|  | Model 1 | -2.39 (-4.69, -0.08) |  | 3.12 (0.17, 6.07) | -0.64 (-3.63, 2.35) | -2.43 (-6.78, 1.93) | 4.00 (0.19, 7.80) |
|  | Model 2 | -2.26 (-4.64, 0.11) |  | 2.94 (-0.08, 5.96) | -0.32 (-3.40, 2.76) | -1.64 (-6.07, 2.79) | 3.52 (-0.37, 7.41) |
|  | Supp |  |  | 2.86 (-0.09, 5.82) | 0.20 (-2.93, 3.33) | -1.95 (-6.32, 2.41) | 3.33 (-0.52, 7.18) |
| Mental health | Crude | -0.97 (-1.91, -0.03) |  | 1.55 (0.27, 2.82) | -1.68 (-2.99, -0.36) | -0.41 (-2.30, 1.48) | 1.95 (0.28, 3.62) |
|  | Model 1 | -0.81 (-1.88, 0.27) |  | 1.11 (-0.27, 2.49) | -2.28 (-3.68, -0.89) | -1.04 (-3.07, 1.00) | 1.13 (-0.65, 2.90) |
|  | Model 2 | -0.91 (-2.01, 0.20) |  | 0.89 (-0.51, 2.30) | -1.96 (-3.40, -0.52) | -0.57 (-2.64, 1.50) | 0.90 (-0.91, 2.71) |
|  | Supp |  |  | 0.83 (-0.54, 2.21) | -1.95 (-3.41, -0.49) | -0.62 (-2.65, 1.41) | 0.87 (-0.91, 2.66) |
| PCS | Crude | -1.07 (-1.70, -0.44) |  | -0.07 (-0.93, 0.79) | -1.25 (-2.14, -0.37) | -0.88 (-2.15, 0.38) | 0.47 (-0.65, 1.59) |
|  | Model 1 | -1.09 (-1.81, -0.38) |  | -0.36 (-1.28, 0.56) | -0.99 (-1.92, -0.06) | -0.67 (-2.02, 0.67) | 0.12 (-1.06, 1.31) |
|  | Model 2 | -0.72 (-1.36, -0.07) |  | -0.19 (-1.01, 0.64) | -0.68 (-1.53, 0.16) | -0.29 (-1.50, 0.91) | 0.08 (-0.98, 1.14) |
|  | Supp |  |  | -0.25 (-1.07, 0.57) | -0.40 (-1.26, 0.47) | -0.52 (-1.72, 0.68) | -0.07 (-1.14, 0.99) |
| MCS | Crude | -0.33 (-0.87, 0.21) |  | 1.07 (0.33, 1.80) | -0.29 (-1.05, 0.46) | -0.07 (-1.14, 1.00) | 1.34 (0.39, 2.30) |
|  | Model 1 | -0.42 (-1.04, 0.19) |  | 0.90 (0.10, 1.69) | -0.69 (-1.49, 0.11) | -0.59 (-1.75, 0.57) | 0.92 (-0.10, 1.94) |
|  | Model 2 | -0.46 (-1.10, 0.17) |  | 0.82 (0.01, 1.63) | -0.49 (-1.32, 0.33) | -0.47 (-1.65, 0.71) | 0.87 (-0.17, 1.91) |
|  | Supp |  |  | 0.80 (0.01, 1.59) | -0.48 (-1.31, 0.36) | -0.49 (-1.65, 0.67) | 0.90 (-0.13, 1.92) |
|  |  |  |  |  |  |  |  |
